# Formal social support and quality of life of caregivers with autistic children: a large-scale nationwide survey in China

**DOI:** 10.3389/fpubh.2023.1282778

**Published:** 2023-12-06

**Authors:** Qingqing Ran, Liangzhi Tu, Yinghui Wu, Shunming Zhang, Erliang Zhang, Huilun Li, Ya Su, Mi Xiang

**Affiliations:** ^1^International Peace Maternity and Child Health Hospital, School of Medicine, Shanghai Jiao Tong University, Shanghai, China; ^2^School of Public Health, Shanghai Jiao Tong University, Shanghai, China; ^3^College of Stomatology, Shanghai Jiao Tong University School of Medicine, Shanghai, China; ^4^School of Nursing, Shanghai Jiao Tong University, Shanghai, China; ^5^Shanghai American School, Shanghai, China

**Keywords:** caregivers, social support, quality of life, children, autism spectrum disorder (ASD)

## Abstract

**Introduction:**

Caregivers of children with autism spectrum disorder (ASD) were reported poor quality of life (QOL). Formal social support might improve the QOL of caregivers, however, limited research to date has focused on this association in China and formal social support for this group is conspicuously lacking. The study was aim to understand the QOL in male and female caregivers with ASD children in China and to explore the relationship between QOL and formal social support for caregivers with children with ASD through a large-scale nationwide survey.

**Methods:**

An online questionnaire was used to conduct a cross-sectional study with a sample of 6,120 caregivers of children with ASD. Relationship between Quality of Life and several potential predictors is measured and analyzed. Quality of life were measured by Medical Study Short-Form Health Survey version 2 (Chinese version). Multivariate logistic regression analysis was used to analyze the factors affecting caregivers' QOL.

**Results:**

The results revealed that the QOL of caregivers of autistic children in China was poor especially male caregivers. Social support was a positive predictor. More importantly, formal social support from rehabilitation institutions can improve caregivers' physical QOL. Caregivers' satisfaction with the rehabilitation institutions affecting their physical and mental QOL.

**Conclusion:**

The formal social support provided by rehabilitation institutions plays a positive role in improving the quality of life of caregivers.

## 1 Introduction

Autism spectrum disorder (ASD) is a life-long pervasive developmental disorder characterized by differences in social interaction, communication, or repetitive behaviors (1). The global prevalence of ASD has increased rapidly over the past few years, ranging from 1.09/10,000 to 436.0/10,000 in 2012 (2). In China, the prevalence of ASD is 1% of the total population, or approximately 13 million people (3). Recently, the increasing community awareness and public health responses have revealed more unidentified and undiagnosed cases (4). The pervasive and severe differences present in ASD children create substantial burdens on society and families.

Caregivers of children with ASD have reported lower physical and mental quality of life (QOL) (5, 6). However, the QOL of male caregivers remains inconclusive since most previous studies solely investigated female caregivers. Among the predictors of QOL of caregivers, social support positively correlates with family-related QOL and may act as a stress buffer when caring for ASD children (7). According to previous research studies (8, 9), social support may be categorized into informal social support, referring support provided by family members and close friends, and formal social support, referring support provided by professionals, such as doctors, psychologists, social workers, governments, non-profit institutions, community groups, and other assistance. A large number of caregivers have reported insufficient understanding and knowledge of the disability, indicating their need for health education and formal social support (10). Nevertheless, the field of autism in China remains underexplored compared with other developed countries and social support for children and their caregivers is inadequate (11). Additionally, only a few studies examined formal social support in caregivers of children with autism in China, while the rest focused on informal social support.

In China, ASD children's caregivers are likely to experience substantial psychological stress in the care process due to social discrimination and self-blame (12). Moreover, the ASD diagnosis, interventions, and support system have not yet been fully established in China (13), limiting available social support to caregivers of autistic children in China. Rehabilitation institutions, which may be an important part of formal social support in China, are found to contribute to an increase in parental knowledge of ASD and better parental confidence as well as a significant reduction in parental anxiety in Western countries (14, 15). On the other hand, rehabilitation institutions are essential in managing challenging behaviors of autistic children, providing support for families with autistic children, and reducing the mental burdens of caregivers by developing personalized diagnosis and treatment programs.

However, the medical and healthcare costs are significantly higher in individuals with ASD than in the general population (16). The annual medical costs for children with ASD were 3767.38 RMB in 2011 (17). The high cost of autism treatment is not covered by the healthcare system in China, while formal social support from the community is usually unavailable to caregivers (18). Financial support from the government reduces the financial burden of caregivers and improves quality of life (19).

The economic and social support for caregivers of children with autism is limited, and there is also a lack of attention from society to the quality of life of this group. Thus, caregivers of children with ASD urgently require financial and professional social support. However, to the best of our knowledge, there is no large-scale research in China exploring the impact of formal social support, particularly support from rehabilitation institutions on the quality of life in autistic children's caregivers.

Therefore, the aim of this study was to understand the QOL in male and female caregivers with ASD children in China and to explore the relationship between QOL and formal social support in caregivers with children with ASD.

## 2 Methods

### 2.1 Study design

This cross-sectional study recruited participants from July 2021 to September 2021. An online survey was delivered to the study participants via Erkang Assistant (Shanghai Muyue Information Technology Development Co., LTD), an online learning platform developed by the Child Rehabilitation Professional Committee of China Life Care Association. Erkang Assistant was the first professional intelligent rehabilitation service provider of “Internet + rehabilitation education for special children” in China. Adopting the trinity operation mode of “platform + community + family,” Erkang Assistant created comprehensive, integrated, high-quality services such as education consultation, evaluation, and training. Through the learning platform of Erkang Assistant, we found WeChat groups (China's Tencent Holdings LTD), such as “Rehabilitation Institution Intervention Group.” The WeChat group members consisted of caregivers of autistic children and rehabilitation personnel in institutions. Informed consent was provided to the participants before proceeding to the survey. All participants had read and chosen the option presenting agreement with the consent before answering any question. Survey links were distributed to the WeChat groups for caregivers to complete voluntarily. At the same time, the survey link was posted on Erkang Assistant's official channel after being reviewed and approved by the platform operator. Each WeChat ID was only allowed to fill out the survey once. Filled data was exported, checked, and cleaned by professionals. All experimental procedures were approved by the local ethics committee and were in accordance with the Declaration of Helsinki. This study was approved by the Ethics Committee of Shanghai Jiao Tong University School of Medicine (SJUPN- 201813).

### 2.2 Participants

According to the age criteria set by the United Nations Educational, Scientific, and Cultural Organization, this study defined children as those belonging to the period from birth to 18 years. We recruited study participants from 31 provinces/autonomous regions/municipalities in China by non-probability sampling. The initial sample included 8,940 participants. The inclusion criteria were as follows: (1) caregivers aged 22–60 years and (2) caregivers of ASD children under the age of 18 years. After excluding incomplete data, missing data, wrong fillings, and outliers, the final sample consisted of 6,120 participants, corresponding to a 68.3% response rate.

The questionnaire screening criteria are as follows:

(1) According to the question “What is your child's current age?” The answer to the questionnaire is invalid if the number is too large, too small, or not a number.
(2) According to the question “What form of welfare do you expect?” the answer that is not a reasonable form of welfare is invalid.
(3) According to the question “What is the economic subsidy you expect to receive per month?” if the answer is unreasonable and the subsidy is not economic, it is invalid.
(4) According to the question “How old are you?” (ask the caregivers' age).
(5) Filling out questionnaires that are <20 years old and are calculated to have children between the ages of 10 and 20 is invalid.

### 2.3 Measures

#### 2.3.1 Demographic characteristics

The age, gender, educational attainment level, and marital status of the caregivers (either male or female), as well as the age and gender of the ASD child were collected (Socioeconomic status was not included. We collected the regions of the fillers and grouped them by regional GDP to understand the overall economic situation).

#### 2.3.2 Caregivers' quality of life (SF-12)

Quality of life among caregivers of children with autism was assessed using Medical Study Short-Form Health Survey version 2 (Chinese version) (SF-12v2), a simplified version of SF-36. The SF-12v2 included 12 items to assess eight health concept subscales: general health (GH), physical functioning (PF), role-physical (RP), bodily pain (BP), vitality (VT), social functioning (SF), role-emotional (RE), and mental health (MH). The physical component summary (PCS) was assessed according to GH, PF, RP, and BP, while the mental component summary (MCS) was assessed according to SF, RE, MH, and VT. The US normal-based standard scores were calculated using guidelines provided by Ware et al. (20). The MCS12 and PCS12 demonstrated high internal consistency (Cronbach's alpha and Mosier's alpha > 0.8) (21). The effect size differences between the standard SF-36 and SF-12 scores were <0.3, showing comparable validity of SF-12 in the Chinese population (22).

According to the definition of Ware et al. (23), the cutoff point of caregivers' QOL score was 50, with a score ≥50 showing caregivers' higher QOL. A higher score indicated a better QOL of the caregiver. Cronbach's α of this study is 0.876.

#### 2.3.3 Social support

Social support was classified into formal social support and informal social support. Informal social support was evaluated by one question asking about whether the caregiver has a partner. Formal social support was further divided into two dimensions. One referred to support provided by the social and government, which was assessed by three questions: (1) Have you ever received help from a rehabilitation facility? (2) Have you ever received any benefits from the government, community, or organization? (3) Do you know the available financial or welfare support in the local community? The other dimension referred to support received from the rehabilitation facility. Five questions were asked, including caregivers' satisfaction with the professionalism and service standardization, service attitude, cost performance and rehabilitation effect of the rehabilitation institution, as well as whether the organization provided caregivers with professional training or guidance.

### 2.4 Statistical analyses

**Figure 1:**
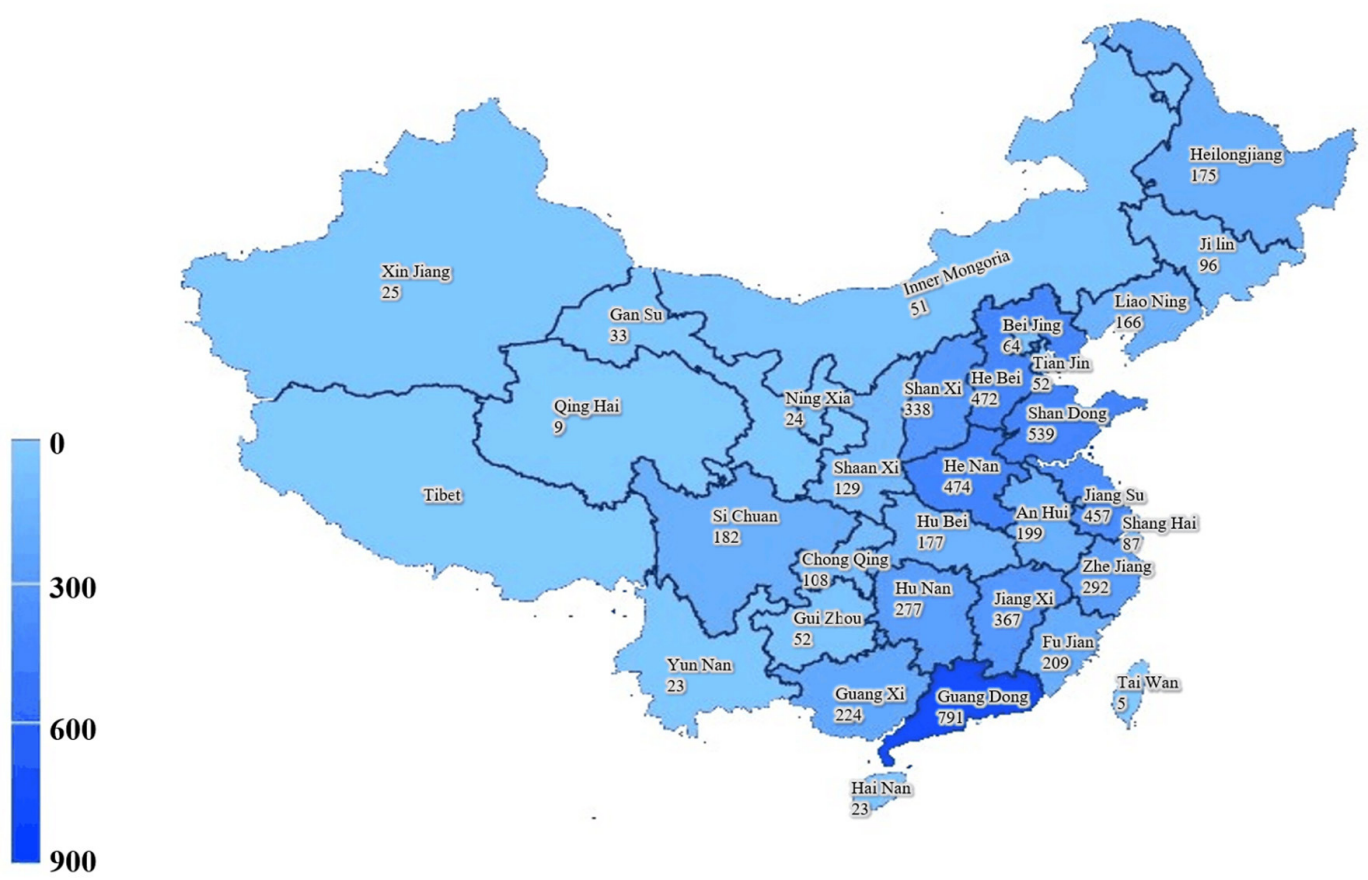
Geographic distribution of participants in China.

A distribution map of the study participants was generated. The density of caregivers was demonstrated by different colors, with darker colors indicating higher density (Figure 1). Descriptive statistics of the demographic characteristics were performed using SPSS26.0 (International Business Machines Corporation).

After conducting the Shapiro normality testing, the PCS and MCS components of the Autism Quality of Life (SF-12) questionnaire were presented in medians and interquartile range (IQR). Categorical data were expressed as frequency (*n*) and percentage (%).

The steps to calculate quality of life using the SF-12 scale are as follows:

(1) Confirm whether there are missing values and whether the score range for each item is correct (the lowest score is calculated from 1).
(2) Correct the reverse scoring to ensure that the higher the score for each item, the better the health status it represents.
(3) Convert the scores of eight dimensions.
(4) Standardize the scores of eight dimensions (Z-score).
(5) Calculate the final score: [(standardize the score ^*^ coefficients of each dimension)] ^*^ 10 + 50.

Univariate logistic regression analysis was performed with PCS and MCS as outcome variables. A preliminary study was conducted to investigate the impact of demographic characteristics and social support of 6,120 caregivers and their children on their quality of life, and P and OR values were obtained. Because the QOL of caregivers was generally low, the majority of caregivers scored below 50 points, indicating an extreme imbalance according to 50 points. The median was used for grouping, preliminarily analyzing the impact of demographic characteristics and social support on the QOL of caregivers.

After collinearity diagnostics, the explanatory variable with the largest variance inflation factor was selected and eliminated, leaving two options representing formal social support at last. One was whether they received welfare from the government or community, and the other question asked whether they had found rehabilitation institutions. Forced logistic regression was used to further explore the possible effects of formal social support from the government and community and from rehabilitation institutions on the QOL of caregivers.

Logistic regression analyses on caregivers who accessed rehabilitation institutions were conducted to understand formal social support further. Independent variables were entered in the following order: factors related to rehabilitation institutions were added in step 1 and step 2 added social-demographic factors for adjustment. Because gender was an important influencing factor in this study, the Appendix provided information on how social support from the rehabilitation institutions affected the QOL of caregivers by gender. The level of statistical significance was set at a *p*-value of < 0.05. All the models were tested by the Hosmer and Lemeshow test model goodness of fit, and all *p*-values were >0.05.

## 3 Results

This study included a total of 6,120 respondents nationwide, with 53.5% male caregivers and 46.5% female caregivers. Figure 1 shows the regional distribution of samples in this study, with each province and the number of samples marked separately and segmented with blue shading.

Table 1 shows the demographic characteristics of the study participants. The median PCS and MCS scores were 39.67 (36.17, 45.16) and 37.63 (35.61, 41.68) respectively. A vast array of participants reported a low level of physical QOL (88.9%) and mental QOL (97.4%) when grouped by 50. The gender and age of children did not influence the caregivers' QOL.

**Table 1:** Descriptive statistics of the study participants (*N* = 6,120) and intragroup demographic comparisons.

**Variable**	***N* (%)**	**PCS**^**a**^ **score**			**MCS**^**b**^ **score**		
		**Median (Q1, Q3)**	***P-*** **value**	***OR***	**Median (Q1, Q3)**	***P*** **-value**	**OR**
**Demographic characteristics of children with autism**							
Age, years			0.398	1.005		0.910	0.999
**Sex**							
Female	2,215 (36.2%)	39.63 (36.07, 45.11)		Ref	37.72 (35.60, 42.10)		Ref
Male	3,905 (63.8%)	39.70 (36.22, 45.19)	0.715	0.981	37.61 (35.62, 41.51)	0.770	1.016
**Demographic characteristics of caregivers**							
Age, years			0.440	0.996		0.011	0.988
**Sex**							
Female	2,844 (46.5%)	39.97 (36.28, 45.56)		Ref	37.89 (35.65, 42.25)		Ref
Male	3,276 (53.5%)	39.48 (36.02, 44.77)	0.026	1.121	37.46 (35.60, 41.30)	0.036	1.114
**Education level**							
Lower than bachelor's degree	2,520 (41.2%)	39.40 (36.09, 44.49)		Ref	37.50 (35.48, 41.11)		Ref
Bachelor's degree and above	3,600 (58.8%)	39.86 (36.22, 45.78)	0.024	0.889	37.78 (35.83, 42.17)	0.253	0.942
**Location (GDP per capita)**							
Q1: < 55,131	1,714 (28.0%)	39.66 (35.97, 45.59)		Ref	37.80 (35.86, 42.07)		Ref
Q2:55,131–63,426	1,405 (23.0%)	39.53 (35.95, 44.95)	0.644	1.034	37.61 (35.47, 41.51)	0.644	1.034
Q3:63,426–88,210	1,701 (27.8%)	39.70 (36.32, 45.13)	0.850	0.987	37.69 (35.88, 41.79)	0.799	1.018
Q4: 88,210–195,800	1,400 (21.2%)	39.77 (36.26, 44.81)	0.653	0.967	37.54 (35.45, 41.46)	0.488	1.052
**Informal social support** ^c^							
**Marital status**							
With partner	5,935 (97.0%)	39.77 (36.24, 45.25)		Ref	37.66 (35.62, 41.76)		Ref
No partner	185 (3.0%)	37.51 (34.19, 40.56)	< 0.001	2.243	37.21 (35.38, 40.43)	0.332	1.156
**Formal social support** ^d^							
**Ever found help rehabilitation facility**							
Yes	1,234 (20.2%)	40.36 (36.76, 46.24)		Ref	37.81 (35.35, 41.67)		Ref
No	4,886 (79.8%)	39.41 (35.95,44.83)	< 0.001	1.390	37.61 (35.67, 41.72)	0.610	1.033
**Ever received any benefits from the government, community or organization**							
Yes	1,228 (20.1%)	40.33 (36.89, 46.37)		Ref	37.88 (35.25, 41.99)		Ref
No	4,892 (79.9%)	39.42 (35.95, 44.77)	< 0.001	1.364	37.60 (35.71, 41.63)	0.407	1.054
**Know financial or welfare support in your community**							
Yes	1,104 (18.0%)	40.45 (36.84, 46.62)		Ref	38.11 (35.76, 42.35)		Ref
No or unclear	5,016 (82.0%)	39.43 (35.98, 44.77)	< 0.001	1.388	37.54 (35.60, 41.59)	0.020	1.168
**Quality of life**		39.67 (36.17, 45.16)			37.63 (35.61, 41.68)		
Low PCS scores (< 50)	5,442 (88.9%)						
Low MCS scores (< 50)	5,963 (97.4%)						

^a^PCS, Physical Component Summary score, includes questions concerning general health (GH), physical functioning (PF), role-physical (RP), bodily pain (BP). ^b^MCS, Mental Component Summary score, includes questions concerning vitality (VT), social functioning (SF), role-emotional (RE), and mental health (MH). OR, Odd ratio; ^c^Informal social support: support provided by family members or close friends. ^d^Formal social support: support provided by professionals, such as doctors, psychologists, social workers, governments, non-profit institutions, community groups, and other assistance.

A significantly lower PCS score (*p* = 0.026, *OR*: 1.121) and lower MCS score (*p* = 0.036, *OR*: 1.114) were reported in male than in female subjects. Caregivers who received a bachelor's degree and above indicated a higher physical QOL than caregivers with a degree lower than a bachelor's degree. The vast majority of participants who had partners (97.0%) showed significantly higher PCS scores (*p* < 0.001, *OR*: 2.243) than participants without partners. No disparities were observed among participants living in different GDP regions. More than half of the participants indicated they never received help from the rehabilitation facility (79.8%), never received any benefits from the government, community, or organization (79.9%), and did not know the available financial or welfare support in the community (82.0%). All of the three variables were closely related to caregivers' physical QOL (*p* < 0.001, *OR*: 1.390; *p*<*0.001, OR*: 1.364; *p* < 0.001, *OR*: 1.388).

Caregivers who knew financial or welfare support in the community were illustrated a higher mental score than caregivers who did not know or unclear.

The associations of the independent and dependent variables were examined by logistic regression.

**Figure 2:**
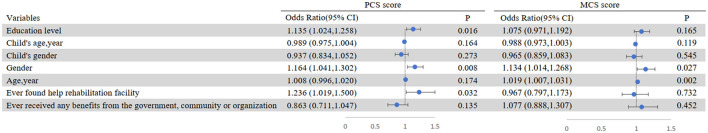
Relationship between demographic information and caregivers' quality of life.

Figure 2 shows the results of caregivers' QOL in PCS and MCS, respectively. Male caregivers were one or two times more likely to have poorer physical QOL (*OR* = 1.164, *CI*: 1.041–1.302, *p* = 0.008) and were less likely to have poorer mental QOL (*OR* = 1.134; CI: 1.014–1.268, *p* = 0.027). Higher-educated caregivers had higher PCS scores (*OR* = 1.135, *CI*: 1.024–1.258, *p* = 0.016) than lower-educated caregivers.

Caregivers who did not seek support from the rehabilitation facilities had a 1.236 times higher risk of poor physical QOL than caregivers who assessed rehabilitation facilities (*P* = 0.032). Receiving welfare from the government, community, or organization did not have a statistically significant effect on caregivers' physical or mental QOL (*OR* = 0.863, *CI*: 0.711–1.047 *p* = 0.135; and *OR* = 1.077; CI: 0.888–1.307, *p* = 0.452, respectively).

Table 2 shows the results of the binary logistic regression model with PCS and MCS as dependent variables for caregivers who had selected rehabilitation institutions.

**Table 2:** Association of formal social support from rehabilitation institutions with caregivers' quality of life.

**Variables**	**PCS** ^**a**^				**MCS** ^**b**^			
	***P*** **-value**	**cOR (95%CI)**	***P*** **-value**	**aOR (95%CI)**	***P*** **-value**	**cOR (95%CI)**	***P*** **-value**	**aOR (95%CI)**
Evaluate the professionalism and service standardization of rehabilitation personnel	0.880	1.033 (0.676, 1.579)	0.833	1.047 (0.684, 1.602)	0.624	0.905 (0.606, 1.351)	0.589	0.895 (0.598, 1.339)
Evaluate the service attitude of rehabilitation personnel	0.011	1.763 (1.140, 2.726)	0.011	1.758 (1.136, 2.721)	0.047	1.513 (1.006, 2.276)	0.052	1.501 (0.997, 2.260)
Evaluate the cost performance of your chosen institution	< 0.001	1.904 (1.367, 2.652)	< 0.001	1.862 (1.334, 2.600)	0.295	1.185 (0.862, 1.630)	0.291	1.189 (0.863, 1.638)
Evaluate the rehabilitation effect of your child in the institution	0.007	1.540 (1.126, 2.106)	0.011	1.510 (1.101, 2.071)	0.017	1.447 (1.069, 1.960)	0.013	1.476 (1.086, 2.005)
Whether the organization provide you with professional training or coaching	< 0.001	2.706 (1.577, 4.642)	< 0.001	2.617 (1.524, 4.496)	0.585	1.136 (0.719, 1.796)	0.552	1.150 (0.726, 1.822)

cOR, crude odds ratio; aOR, adjust odds ratio; CI, Confidence interval. ^*a*^PCS, Physical Component Summary score, includes questions concerning general health (GH), physical functioning (PF), role-physical (RP), bodily pain (BP). ^*b*^MCS, Mental Component Summary score, includes questions concerning vitality (VT), social functioning (SF), role-emotional (RE), and mental health (MH). Adjusted by children's age, children's sex, caregivers' sex, caregivers' age, caregivers' education level, and marital status.

After adjusting demographic variables, those who were dissatisfied with the service attitude of the rehabilitation institution had 1.758 (*p* = 0.011) times lower physical QOL. Caregivers who thought the institution was not cost-effective had 1.862 (*p* < 0.001) times lower physical QOL. Caregivers who were dissatisfied with the rehabilitation effect of the child had 1.510 (*p* = 0.011) times lower physical QOL. Caregivers unclear or did not receive welfare or support for autistic families provided by the community had 2.617 (*p* < 0.001) times lower physical QOL. Caregivers who were dissatisfied with the effectiveness of the child's rehabilitation facility were 1.476 (*p* = 0.013) times more likely to have a lower psychosocial composite score. These results confirmed that formal social support from rehabilitation institutions had an impact on caregivers' QOL, and there were different factors influencing the two dimensions.

Since gender is an important influencing factor, we classified them according to gender to explore the relationship between male and female caregivers' satisfaction with rehabilitation institutions and their quality of life. Full details are given in the Appendix (refer to Tables 3, 4 in Appendix).

## 4 Discussion

In this article, the QOL of caregivers with ASD children in China is examined, and the relationship between QOL and formal social support is explored. The findings indicate that the QOL of caregivers is generally low. Male caregivers, caregivers without partners, and caregivers with low education levels are significantly associated with poor physical QOL. Our analysis did not detect any association between community/government support and caregivers' QOL. In contrast, support from rehabilitation facilities is significantly associated with low QOL.

Regarding caregivers' characteristics, the current study discovers a more impaired QOL in male caregivers than in female caregivers. Most previous studies reported a lower QOL, higher physical pain, and higher levels of fatigue and tiredness in female caregivers than in male caregivers (24–26). Nevertheless, the findings of our study are not allied with these results. One possible explanation is the large proportion (53.5%) of male participants recruited in our study. Male caregivers are rarely examined in previous studies, and the sample size is usually small, resulting in inaccurate and non-representative findings. On the other hand, recent changes in social expectations may lead to variations in the findings. In China, the traditional social expectations of women taking the primary caregiving role (27) have shifted in recent decades due to the changes in family structure, diversification of the family model, and empowerment of females. As men become more involved in the caregiving process, the burdens of caring for children, particularly challenged children, are more shared by both male and female caregivers. Moreover, Chinese men may bear more economic burden when caring for ASD children since men are commonly assumed to have higher social and financial responsibility than women in China, which further deteriorates their QOL. Our results not only underline the significance of interfering with the QOL of caregivers with autistic children in general but also uncover the urgency of QOL interventions targeting male caregivers.

Caregivers of ASD children receive lower social support compared with caregivers with healthy children (28) possibly due to their avoidance of social support-seeking behavior (29), negatively influencing their QOL. Several studies (30, 31) have highlighted the effect of informal social support on the QOL, which may support caregivers with inadequate formal support services to some extent (32). In accordance with previous studies, marital status, one indicator of informal social support, significantly affects the caregivers' PCS score in our study, suggesting caregivers with partners may feel less physical isolation and helplessness when caring for ASD children. Our study advocates the positive effect of spouse support on the psychological level found in earlier research and can be used as a supplement.

Furthermore, formal social support also has a significant impact on caregivers' QOL, which is consistent with previous studies (33–35). In our study, formal social support provided by the government and the community is not associated with the QOL of caregivers. It may indicate inadequate welfare for autistic families in most areas at present, or the services and benefits are not readily accessible (36). However, the practical significance of such support may be masked because 5,016 (82.0%) caregivers in this study did not know or accept the community benefits for autistic families. On the one hand, the limited content of our questionnaire may not cover all types of support provided by the government and the community. A team in the United States has established a mobile platform called “GapMap” to display the accessibility and availability of autism-related resources (37), which provides approaches for autistic families to assess social support and is worth our reference.

A significant proportion of caregivers who reached rehabilitation institutions has reported that they lacked the knowledge and understanding of the disability compared to healthcare professionals (38), and caregivers feel frustrated when they are not competent at caring (39), underlying caregivers' need of health and disease related-education. Establishing a partnership between caregivers and the rehabilitation facility may allow healthcare providers to deliver personalized rehabilitation plans to meet caregivers' needs (40).

The cost-effectiveness of rehabilitation institutions significantly correlated with caregivers' QOL. Families with ASD children spend a substantial amount of time, effort, and money raising their children, especially in China (41). The services and attitude of the personnel in the rehabilitation institutions may directly affect the trust between caregivers and healthcare providers by affecting the rehabilitation effectiveness, which may influence the caregivers' physical QOL. Therefore, the healthcare professionals of rehabilitation institutions should be well trained in coping with different ASD children to achieve a higher level of rehabilitation effect, which is an important way to comfort the QOL of caregivers.

In our study, the rehabilitation efficacy is particularly critical to the mental health domain of caregivers' QOL. Although the construction of a formal social support system for autistic children has been prioritized at national and local levels in China to date, the service standards and related policies for autism rehabilitation or managed care institutions are still insufficient (18). The establishment of a standard qualification for the rehabilitation personnel may improve the treatment effectiveness and quality of the psychological life of caregivers. Future development in China should emphasize the specialized services for the families of ASD children (42).

Some limitations should be considered when interpreting our results. First of all, reporting economic status is not mandatory since family economic status is considered private, which results in multiple missing values. Consequently, family economic status is not included in our analysis. Second, more than 90% of the caregivers reported having partners, which may require more research to reveal the impact of informal social support on caregivers' QOL. In addition, this study was carried out during the COVID-19 pandemic, which may have a certain influence on the QOL of the caregivers. However, the pandemic was well controlled during the data collection period, lowering the potential impact of COVID-19. Rehabilitation centers may adopt telemedicine, as proposed in another research, to provide more diversified services contracting the influence of COVID (43). Finally, since our study uses a subjective measure of caregivers' evaluation of their QOL, the results may be affected by the social expectations of respondents. Objective indicators of caregivers' QOL may be used in future. This cross-sectional study cannot examine the long-term effect of social support. Thus, future longitudinal studies can focus on the long-term impact of rehabilitation institutions on ASD.

The large nationwide sample of our study has high representativeness and generalizability. Interpretations of the study can be summarized in four-folds: 1. The QOL of male caregivers deserves scrutiny in future studies. 2. Training and education of caregivers' professional knowledge and skills are needed. 3. Policymakers should standardize the service and management of rehabilitation medical centers. 4. Rehabilitation institutions should not only provide professional services to children but also professional support to the parents in a timely manner. The services provided by rehabilitation institutions also need to be more diversified to counteract possible large public crisis, such as COVID-19.

## 5 Conclusion

This nationwide large-scale study discovered a low QOL in Chinese caregivers of autistic children, and the utilization of social support by caregivers of ASD children was unsatisfactory. Both informal and formal social support positively influence the caregivers' QOL. The positive effect of formal social support provided by rehabilitation institutions serves as a basis for autism policy-makers to standardize the rehabilitation institutions. Our study can be used as a reference for improving the QOL of caregivers with autism. We call for more personalized social support to improve caregivers' QOL in future.

## Data availability statement

The original contributions presented in the study are included in the article/Supplementary material, further inquiries can be directed to the corresponding authors.

## Ethics statement

The studies involving humans were approved by the Ethics Committee of Shanghai Jiao Tong University School of Medicine (SJUPN- 201813). The studies were conducted in accordance with the local legislation and institutional requirements. The participants provided their written informed consent to participate in this study.

## Author contributions

QR: Conceptualization, Data curation, Investigation, Methodology, Writing – original draft. LT: Investigation, Writing – original draft. YW: Validation, Writing – review & editing. SZ: Investigation, Writing – review & editing. EZ: Validation, Writing – review & editing. HL: Writing – review & editing. YS: Supervision, Writing – review & editing. MX: Supervision, Writing – review & editing.
