# Supplementary material for: Formal social support and quality of life of caregivers with autistic children: a large-scale nationwide survey in China
Source: Front Public Health. 2023 Dec 6;11:1282778. doi: 10.3389/fpubh.2023.1282778 (PMC10766108; doi:10.3389/fpubh.2023.1282778)
Supplement: Supplementary file 1 [file Table_1.DOCX]

**Appendix:**

| **Table 3 Association of formal social support from rehabilitation institutions with male caregivers' quality of life.** | | | | | | | | | | | | | | | | | |  |
| --- | --- | --- | --- | --- | --- | --- | --- | --- | --- | --- | --- | --- | --- | --- | --- | --- | --- | --- |
| Variables | | PCS scores | | | | | | | MCS scores | | | | | | | | |  |
|  |  | P | cOR(95%CI) | | P | | aOR(95%CI) | | P | | cOR(95%CI) | | P | | aOR(95%CI) | |  |  |
| Evaluate the professionalism and service standardization of rehabilitation personnel | | 0.686 | 0.887(0.497,1.584) | | 0.590 | | 0.851(0.473,1.532) | | 0.535 | | 1.190(0.687,2.060) | | 0.466 | | 1.229(0.706,2.139) | |  |  |
| Evaluate the service attitude of rehabilitation personnel | | 0.249 | 1.420(0.782,2.577) | | 0.264 | | 1.409(0.772,2.572) | | 0.270 | | 1.372(0.783,2.404) | | 0.346 | | 1.312(0.745,2.311) | |  |  |
| Evaluate the cost performance of your chosen institution | | 0.004 | 1.868(1.215,2.870) | | 0.007 | | 1.832(1.184,2.834) | | 0.566 | | 1.128(0.747,1.705) | | 0.560 | | 1.132(0.745,1.720) | |  |  |
| Evaluate the rehabilitation effect of your child in the institution | | 0.003 | 1.876(1.246,2.824) | | 0.004 | | 1.833(1.213,2.769) | | 0.108 | | 1.384(0.931,2.056) | | 0.095 | | 1.406(0.943,2.096) | |  |  |
| Whether the organization provide you with professional training or coaching | | 0.004 | 3.054(1.414,6.595) | | 0.007 | | 2.906(1.340,6.303) | | 0.187 | | 1.542(0.811,2.933) | | 0.227 | | 1.492(0.779,2.855) | |  |  |
| **Table 4 Association of support from rehabilitation institutions with female caregivers' quality of life** | | | | | | | | | | | | | | | | | | |
| Variables |  | | | | |  | | | |  | |  | |  | |  | | |
|  | PCS scores | | | | | | | | | MCS scores | | | | | | | | |
|  | P | | | cOR(95%CI) | | P | | aOR(95%CI) | | P | | cOR(95%CI) | | P | | aOR(95%CI) | | |
| Evaluate the professionalism and service standardization of rehabilitation personnel | 0.88 | | | 0.968(0.633,1.479) | | 0.850 | | 0.955(0.624,1.462) | | 0.477 | | 1.157(0.774,1.730) | | 0.444 | | 1.171(0.782,1.753) | | |
| Evaluate the service attitude of rehabilitation personnel | 0.011 | | | 1.763(1.140,2.726) | | 0.011 | | 1.758(1.136,2.721) | | 0.055 | | 1.491(0.990,2.244) | | 0.060 | | 1.481(0.983,2.232) | | |
| Evaluate the cost performance of your chosen institution | <0.001 | | | 1.904(1.367,2.652) | | <0.001 | | 1.862(1.334,2.600) | | 0.255 | | 1.203(0.875,1.656) | | 0.244 | | 1.210(0.878,1.668) | | |
| Evaluate the rehabilitation effect of your child in the institution | 0.007 | | | 1.540(1.126,2.106) | | 0.010 | | 1.510(1.101,2.071) | | 0.015 | | 1.459(1.077,1.976) | | 0.011 | | 1.487(1.094,2.020) | | |
| Whether the organization provide you with professional training or coaching | <0.001 | | | 2.706(1.577,4.642) | | <0.001 | | 2.617(1.524,4.496) | | 0.657 | | 1.109(0.702,1.754) | | 0.622 | | 1.123(0.708,1.780) | | |
